# Environmental Risk Assessment Strategy for Nanomaterials

**DOI:** 10.3390/ijerph14101251

**Published:** 2017-10-19

**Authors:** Janeck J. Scott-Fordsmand, Willie J. G. M. Peijnenburg, Elena Semenzin, Bernd Nowack, Neil Hunt, Danail Hristozov, Antonio Marcomini, Muhammad-Adeel Irfan, Araceli Sánchez Jiménez, Robert Landsiedel, Lang Tran, Agnes G. Oomen, Peter M. J. Bos, Kerstin Hund-Rinke

**Affiliations:** 1 Department of Bioscience, Aarhus University, Vejlsøvej 25, P.O. Box 314, 8600 Silkeborg, Denmark; 2 National Institute for Public Health and the Environment (RIVM), P.O. Box 1, 3720 BA Bilthoven, The Netherlands; Willie.Peijnenburg@rivm.nl (W.J.G.M.P.); Agnes.Oomen@rivm.nl (A.G.O.); Peter.Bos@rivm.nl (P.M.J.B.); 3 Centre for Environmental Sciences, University Leiden, P.O. Box 9518, 2300 RA Leiden, The Netherlands; 4 Department of Environmental Sciences, Informatics and Statistics, University Ca’ Foscari of Venice, VEGApark, Via delle Industrie 21/8, 30175 Marghera (VE), Italy; Semenzin@unive.it (E.S.); Danail.Hristozov@unive.it (D.H.); marcom@unive.it (A.M.); 5 Swiss Federal Laboratories for Material Science and Technology, EMPA, 8600 Dübendorf, Switzerland; Bernd.Nowack@empa.ch; 6 The REACH Centre, Gordon Manley Building, Lancaster Environment Centre, Lancaster University, Lancaster LA1 4YQ, UK; N.Hunt@thereachcentre.com; 7 Greendecision Srl., Via delle Industrie 21/8, 30175 Marghera (VE), Italy; 8 Experimental Toxicology and Ecology, BASF SE, RB/TB-Z470, 67056 Ludwigshafen, Germany; muhammad-adeel.irfan@basf.com (M.-A.I.) robert.landsiedel@basf.com (R.L.); 9 Institute of Occupational Medicine, Centre for Human Exposure Science (CHES), Research Avenue North, Riccarton, Edinburgh EH14 4AP, UK; Araceli.Sanchez@iom-world.org (A.S.J.); lang.tran@iom-world.org (L.T.); 10 Fraunhofer Institute for Molecular Biology and Applied Ecology, Auf dem Aberg 1, 57392 Schmallenberg, Germany; Kerstin.Hund-Rinke@ime.fraunhofer.de

**Keywords:** nanomaterials, environment, risk assessment

## Abstract

An Environmental Risk Assessment (ERA) for nanomaterials (NMs) is outlined in this paper. Contrary to other recent papers on the subject, the main data requirements, models and advancement within each of the four risk assessment domains are described, i.e., in the: (i) materials, (ii) release, fate and exposure, (iii) hazard and (iv) risk characterisation domains. The material, which is obviously the foundation for any risk assessment, should be described according to the legislatively required characterisation data. Characterisation data will also be used at various levels within the ERA, e.g., exposure modelling. The release, fate and exposure data and models cover the input for environmental distribution models in order to identify the potential (PES) and relevant exposure scenarios (RES) and, subsequently, the possible release routes, both with regard to which compartment(s) NMs are distributed in line with the factors determining the fate within environmental compartment. The initial outcome in the risk characterisation will be a generic Predicted Environmental Concentration (PEC), but a refined PEC can be obtained by applying specific exposure models for relevant media. The hazard information covers a variety of representative, relevant and reliable organisms and/or functions, relevant for the RES and enabling a hazard characterisation. The initial outcome will be hazard characterisation in test systems allowing estimating a Predicted No-Effect concentration (PNEC), either based on uncertainty factors or on a NM adapted version of the Species Sensitivity Distributions approach. The risk characterisation will either be based on a deterministic risk ratio approach (i.e., PEC/PNEC) or an overlay of probability distributions, i.e., exposure and hazard distributions, using the nano relevant models.

## 1. Introduction

Concern has been raised regarding whether engineered Nanomaterials (NMs) cause environmental harm. Further, it is already realised that at least some elements of the present regulatory risk assessment (RA) approach are not adequate to reflect NM risk. The inadequacy includes, for example, an insufficient description of the relevant material characteristics (e.g., as these must be used in fate and exposure models), a lack of relevant exposure models (e.g., the present models do not take NM behaviours into account), a lack of knowledge on which species are mostly affected by NMs (e.g., the present approaches prioritise aquatic pelagic organisms, whereas for NMs the organisms most likely affected are terrestrial), and how to include such in risk characterisation (e.g., there is presently no way to account for NM relevant parameters). Based on this concern and insight, novel tools and approaches to evaluate NM risk have been suggested, as reviewed by [1,2]. Various conceptual frameworks have been outlined by [3]—a meta assessment approach, [4]—a general human and environmental approach focusing on limited testing, [5]—a general overview of the policy related information, and [6]—with a more flexible and integrating exposure drive RA approach. Here, we describe an Environmental Risk Assessment (ERA) strategy that, contrary to the previously mentioned approaches, includes the most recent environmental model types in the different domains of risk assessment, i.e., material, exposure, hazard and risk characterisation. This ERA strategy is an exposure driven process comprising two general phases covering the entire life cycle of the material. It is a NM specific adaptation of the MARINA RA strategy presented by [6] to the environment compartment, introducing environment nanospecific issues. 

In summary, in [6] the RA is divided into two phases, Phase 1: the problem framing phase, is (i) based on Potential Exposure Scenarios (PES) to identify Relevant Exposure Scenarios (RES) throughout a NM’s life cycle and (ii) to identify the information required to evaluate whether a specific RES combined with quantitative identified hazards may result in identification of environmental risks. Hence, Phase 1 involves identifying and collecting basic information that should be available for a NM to initiate the risk assessment process and should anticipate the major further requirements in Phase 2. Phase 2: The risk assessment phase aims to provide a targeted RA based on the identified RES [which may be refined] and on the identified information requirements pertaining to NMs’ properties, fate/kinetics, exposure and effects. The specificity of the RA may depend on the user, e.g., industry may need a lower tier RA to design safer NMs, regulators may wish a comparative or a detailed RA, etc.

To optimise resources used in ERA, it may be adequate (when uncertainty is known) to base the ERA (or parts thereof) on grouping and/or read-across approaches. This type of approach can be performed at all steps of the ERA when uncertainty within a step is known or can be estimated. A group represents a number of NMs that share a commonality relevant for risk, which can be one or more common property(ies) in a physical, chemical, exposure, (eco)toxicological, toxicokinetics or fate sense, (see e.g., [7,8,9,10]). It may also be relevant to search online platforms or tools to such obtain information, obviously after sufficient relevance and quality check (e.g., [11,12,13,14]). A NM can belong to more than one group. For further discussion regarding grouping of nanomaterials as proposed by MARINA, (see [15]).

## 2. The Environmental Risk Assessment Strategy

**Figure 1:**
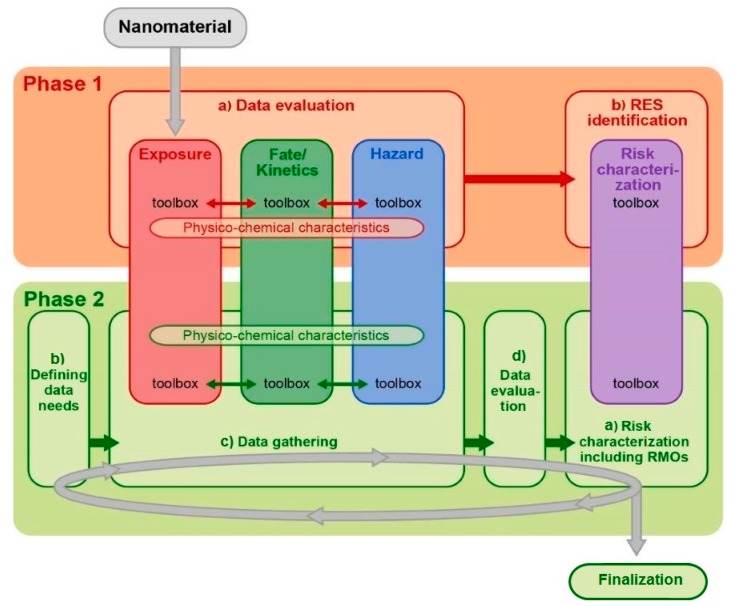
Schematic overview of the MARINA Risk Assessment Strategy, consisting of: (1) an overarching “Phase 1: Problem framing” (orange disc), (2) the iterative “Phase 2: Risk assessment” (green discs: cyclic evaluation process and a finalization step), (3) the three information-gathering pillars: Exposure (red), Fate/Kinetics (green) and Hazard (blue) and (4) the Risk characterization pillar (purple). Phase 1 consists of two steps: (a) Data evaluation, and (b) RES identification. The iterative evaluation process of Phase 2 consists of four steps: (a) Risk characterization including RMOs, (b) Defining data needs, (c) Data gathering and (d) Data evaluation. (Taken from [6]).

The following describes how the MARINA Risk Assessment Strategy proposed by [6] can be implemented for the environment. It is shown how the most recent scientific developments in the material characterisation, release, fate and exposure characterisation, hazard characterisation and risk characterisation domains can be integrated into the ERA. The domains described here correspond to the pillars of the MARINA Risk Assessment Strategy described in [6], see Figure 1. 

Specifically, the release, fate and exposure and the hazard domains that are addressed in the ERA correspond to the three information-gathering pillars (i.e., exposure, fate/kinetics, and hazard) defined by [6]; the “risk characterisation” domain corresponds to the fourth pillar defined by [6] containing tools for the integration of information. The materials domain is not a pillar itself in [6], but it provides information on physico-chemical properties influencing all the four pillars. 

**Figure 2:**
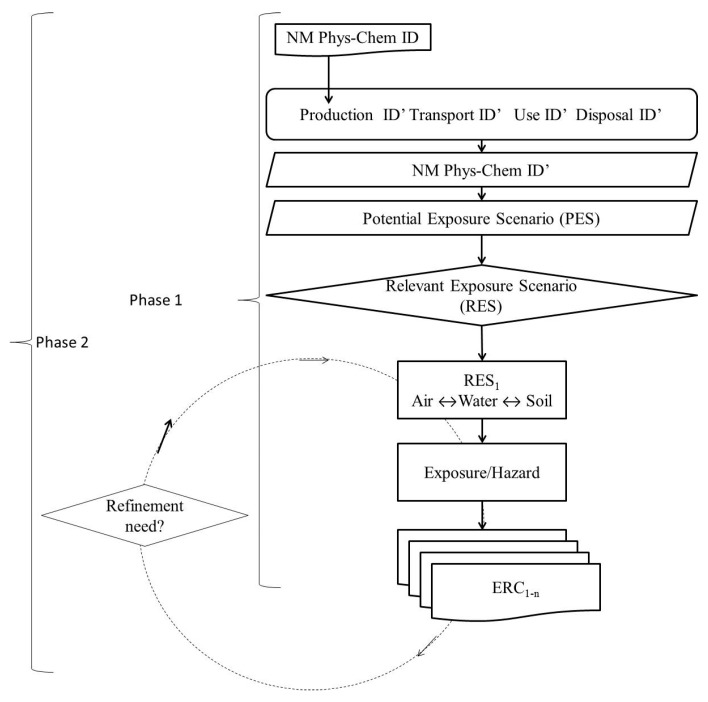
The overall Environmental Risk Assessment (ERA) Strategy.

Hence, the scope of this article is to outline the latest development for each domain and link this into the ERA (see outline Figure 2).

### 2.1. Materials

**Figure 3:**
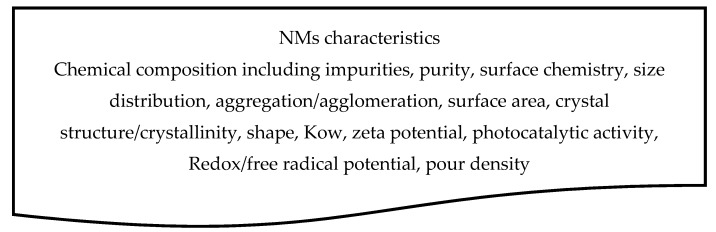
The NMs’ characteristics information, based on reliable measurements, is the starting point for risk assessment.

The materials should be characterised according to the required physicochemical parameters (NMs characteristics, see Figure 3) to enable their identification along their life cycles, to enable exposure and hazard modelling and, if possible, to link to other ERA strategies, e.g., if a NM dissolves, then the risk should in part be assessed for the dissolved species, e.g., metal ions.

The NM parameters should at least include key physicochemical characteristics and properties relevant for release, exposure and hazard testing and modelling (Figure 3, OECD WPMN 41, [15]). Presently, although such physicochemical parameters can be measured for NMs (i), the analytical techniques to do so are not broadly available, (ii) there is still insufficient knowledge concerning the reproducibility of these measurements, (iii) the characterisation is mainly concerned with the pristine form and does not cover the later NM life cycle stages, and (iv) the measurements may in many cases depend on the media that surrounds the NMs (see Section 2.2.3). 

Although the dose-metrics in the final ERA are currently based on mass, the NMs’ characteristics should enable a refined exposure and hazard modelling because environmental distribution, uptake and toxicity will be based on NM specific parameters (see Section 2.2 and Section 2.3). For example, the uptake-rate is to some extent dependent on surface charge, shape and diameter [16] and the environmental distributions are to some extent dependent on surface charge, density, dissolution [17]. As mentioned above, NMs’ characteristics (and properties/behaviour) may also provide a necessary link to other ERA strategies, which will ensure coherence between ERA estimates. Recently, a method was proposed for integrating physico-chemical (PC) characteristics into risk assessment [18].

Finally, ideally reference materials should be used when validating the NM’s characterisation; however, currently almost no nano-sized reference materials are available, exceptions being nanosilicon dioxide from Institute for Reference Materials and Methods (IRMM, European Commission, Geel, Belgium), nanosilver from Bundesanstalt für Materialsforschung (BAM, Berlin, Germany) and nanogold from National Institute of Standards and Technology (NIST, Gaithersburg, MD, USA). The JRC (EC) has set up a repository hosting manufactured nanomaterials, which are representative test materials, (see [19]).

Further, the detailed NM’s characteristics may also be used at all stages in the ERA to group, rank or model NMs for estimating values for data that is missing, e.g., to estimate whether various Ag based NMs (e.g., AgNP, AgONP) can be considered within the same individual risk characterisation. For example, although hazard may not be known for a material, it may be possible to group, rank or model the hazard by comparing it to other materials with similar hazard relevant NM parameters. Suggestions on how to use various NM characeristics in risk ranking have been provided (e.g., [20,21]). 

Lifecycle 

**Figure 4:**
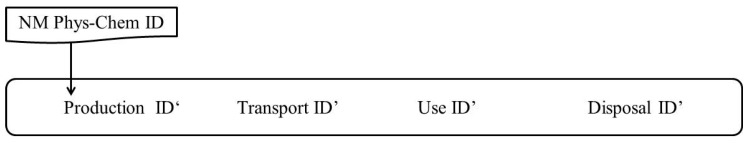
The NMs should be characterised in each point over the entire life cycle, here exemplified by four life cycle steps.

The life cycle of the products containing the NM covers all stages, from material production to end of life, and determines the potential for release [22]. As a first step, four or more main stages of a NM’s life cycle may be identified (Figure 4): production and formulation, transport (i.e., transfer from production to application/use location), application/use and disposal/waste. In each of these stages, all scenarios resulting in exposure of the organisms in the environment to the NMs should be identified. Hence, during its life-cycle the NM may be released to the environment, possibly in a form specific to the life cycle instance at which it is released. After release, transformation of the released NM by for instance dissolution and weathering of the particle, including the coating, should be considered. The amount released and the form of the NM may depend on the instance of the lifecycle and should be estimated. The importance of these changes regarding the impact on environmental distribution, exposure and hazard levels should also be evaluated, e.g., is the NM released at a certain point of the life cycle in different physicochemical forms in such a way that it affects the risk assessment [23]. Such release scenarios may be based on deterministically or probabilistically based mass balance measurements/estimates.

### 2.2. Release, Fate and Exposure

#### 2.2.1. Identification of Exposure Scenarios

**Figure 5:**
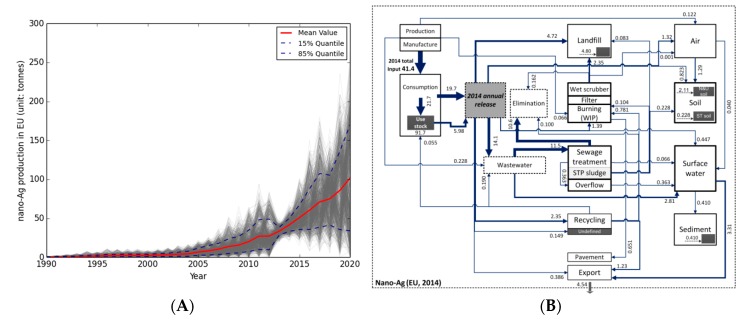
(**A**) and (**B**). Modelled developmental trends for nano-Ag production from 1990 to 2020. The grey curve represents a random result from a probabilistic modelling. The mean values (red line) and the quantiles 0.15 (lower dashed line) and 0.85 (upper dashed line) are also shown. (**B**) Resulting dynamic modelling of mass-flow for nano-Ag in EU for 2014, with the arrow thickness indicating quantity of mass flow.

Based on release scenarios, exposure scenarios can be built [22]. One or multiple exposure scenarios can be described based on information such as the production process, possible down-stream uses, application(s) and use(s) (e.g., in consumer products), and disposal. NMs’ properties should be considered when building exposure scenarios, since NMs’ properties can result in accumulation or leaching and, hence, determine which environmental compartments are exposed and possibly affected. Each exposure scenario can be described qualitatively and/or quantitatively, depending on available information, including at least the potential for release and the exposed environmental compartments (Figure 5). For each RES, the relevant distribution of exposure concentrations within an environmental compartment and the hazard information required are described (see Section 2.2 and Section 2.32.3). 

The RES can be based on various material flow models that are either deterministic or probabilistic distribution models, which identify the contributions to specific environmental compartments in absolute values (e.g., [24]) or as probabilistic distribution (e.g., [25]). 

#### 2.2.2. Models for RES Identification

The selection of the approaches to estimate environmental RES can be based on two principles: first, the expected capability of an approach to represent a system of material flows to predict environmental concentrations and, second, the coverage of a large variety of underlying modelling and simulation mechanisms. The general approach of Material Flow Analysis (MFA) is the tool of choice to model material flows as period-oriented transfer of a material between system entities [26,27]. In MFA, the flows of materials are followed from manufacturing, to use and end of life treatments and transfers to technical and environmental compartments are quantified. MFA has been applied to predict NM flows by various approaches, (e.g., [24,28,29]).

Probabilistic Material Flow Analysis (PMFA) is a modelling approach that was specifically designed to cope with the large uncertainties and variabilities for many input parameters in MFA [30] by extending the classical MFA approach with Bayesian statistics. PMFA describes a stable state in a system of dependent material flows under substantial uncertainties. PMFA has been used to predict flows of several NMs in different regions (Switzerland, EU, US, Australia, and Denmark, [31,32]).

Most of these MFA and PMFA models are top-down models; top-down in this context means that the starting point for the model is the region-wide NM-production, which is then distributed to different product categories. Bottom-up models, on the other hand, (e.g., [24]) start with the uses of consumer products and market penetrations for nano-products. In this way, two complementary approaches have been used that have different data requirements. Based on the available information, different models may be used, considering the fact that they may provide different answers. For example, some models only consider certain applications of the NM (mainly bottom-up models), while others have a comprehensive approach as needed for top-down modelling. Some models also include transformation reactions, mainly during wastewater treatment, but mostly assume that there is no environmental degradation, dissolution, agglomeration or binding of the NMs. Some (early) models assumed that all of the NMs are released from the products and therefore present worst case estimates of PECs, whereas more advanced models derive release factors based on experiments or derive estimates based on expert knowledge.

A further development in MFA is the inclusion of time-dependent aspects, extending MFA and PMFA to a dynamic (P)MFA [33]. Dynamics are most important for the system inflow and the release modelling. NM production increases over time (see Figure 5A) and models for predicting concentrations in environmental sinks such as soils need to consider this increasing input over time. Many NMs are contained in products with a long life-time and a delayed release (e.g., electronics, polymers). In order to accurately predict release from such products, dynamic modelling is needed. Figure 5B shows the resulting mass flows for nano-Ag in 2014, predicted with the dynamic model. 

The MFA models can be used to obtain either generic PEC covering a full media or specific PECs that take into consideration media properties, both based on total values. To arrive at a better understanding of the possible consequences, refined exposure scenarios should be developed (see below).

#### 2.2.3. Identification of Exposure

Once released into the environment, the environmental distribution may be assessed with the fate models (see above), and the within-compartment fate of NMs is determined by several physicochemical processes such as aggregation, sedimentation, particle-deposition, dissolution, etc. [17]. Specific for NMs, these dynamic processes are best described as changes of rates rather than as equilibrium systems. In order to develop new or extend and improve existing models for environmental fate and behaviour of NMs, it is important to understand the processes involved in controlling fate of NMs in the environment and be able to derive quantitative descriptions (e.g., rate constants) of the relevant fate process. The available models, although in their infancy, show different levels of complexity. The exposure bottom-up modelling includes information about important mechanisms affecting the behaviour and fate in the environment, e.g., aggregation, sedimentation and/or dissociation or other relevant processes [34]. Therefore, if possible, knowledge from other natural sciences, such as colloid chemistry or (organic) chemical kinetics and theories dealing with colloids or organic chemicals, should be included. Modelling of processes may be difficult or impossible in the case where no experimental data or relevant values are available, as e.g., the attachment efficiency for different particles to clay and other porous materials.

Alternative approaches are the exposure top-down approaches in which the environmental compartment is treated as a black box and no specific information about the processes occurring within it is normally needed. The drawback of this approach is the limit of predictive potential for new or not tested materials or processes. Hence, this type of model is currently used to e.g., describe the emissions of NMs to the environment and subsequent mobility using partitioning factors, which differentiate between the different fractions, e.g., transported into other compartments or remaining in the water phase. 

Equilibrium partition coefficients, such as the octanol water partition coefficient (Kow) or soil water partition coefficient (Kd), are a powerful tool for the prediction of fate and behaviour of organic chemicals, but are not applicable to NMs due to the fact that most NMs in the environment are present as thermodynamically unstable dispersions. They are, thus, kinetically controlled and do not reach equilibrium, and any coefficient based on equilibrium cannot be used as fate descriptors for NMs [35]. Nonetheless, other descriptors based on the behaviour of NMs can be used to model the environmental fate of NMs, and they are presented below. The specific fate and behaviour of nanoparticles in suspension differ dramatically from those of conventional chemicals. For conventional chemicals, equilibrium partitioning of constituents between the different phases (solid-liquid-gas) of the environmental medium as a result of sorption, solubility and equilibrium chemical reactions is a widely used assumption. When a water-containing contamination is mixed with a solid medium, the constituent mass begins to partition between the solution, the solid and any gas present in the medium [36]. NMs give rise to new challenges for the development and implementation of fate models. Nanoparticles in aqueous dispersions are colloids and are, thus, contrary to chemicals never in thermodynamic equilibrium [37]. As discussed above, the fate of NMs is determined by several processes, which are best described by rates of a change rather than equilibrium. These kinetic aspects of “non-stability” are contrary to the usual assumptions for conventional fate modelling, since the standard multi-media fate models are based on thermodynamic equilibrium that is assumed to occur homogeneously throughout the soil, so that it can be compared with the individual endpoint values obtained by hazard assessments for in homogeneous circumstances. The importance of colloid mediated transport of molecular contaminants has been recognized for many years, but the concept has not yet been integrated in any routine risk-assessment scheme. It is, thus, unclear how spatial and temporal exposure information for NMs can be combined with generic hazard values, providing additional uncertainty. To account for the concentration, there are alternatives. One is to use only the maximum modelled concentration, which is assumed to be the most conservative approach; another is to take the average concentration or integrated exposure into account.

**Figure 6:**
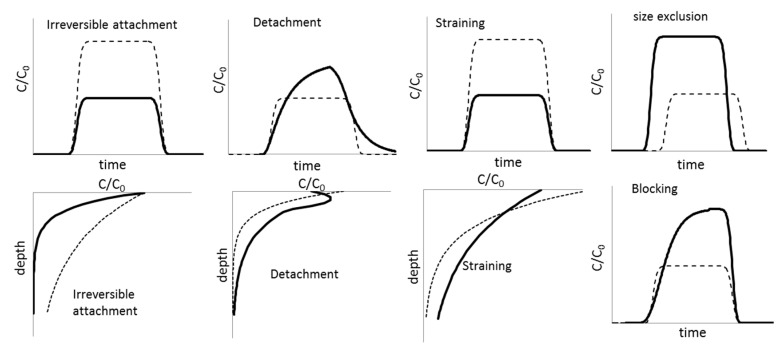
Schematic representations of hypothetical breakthrough curves (column outflow concentrations as a function of time) or depth profiles (solid concentrations as a function of depth). Full lines show how an enhancement of the proposed mechanism could affect results relative to a reference situation where only irreversible attachment occurs in dotted lines, see further [38].

Using predictions of transport modelling is not straightforward for several reasons. NM-modelling in soil has so far been performed by fitting the parameters from Table 1 to breakthrough curves shown in Figure 6, see further [38]. While this procedure has proven extremely helpful to reveal deposition mechanisms, it does not allow predicting concentrations following exposure to NMs in a given soil. Currently, no agreed model framework exists, but the least parameters are required and the most data is available for Colloid Filtration Theory (CFT) (Equations in Table 1). Empirical relations (pedotransfer functions) between α_att_ (attachment efficiency), d50 (detachment constant) and routinely measured parameters such as pH, texture and organic carbon content seem feasible [39,40]. 

CFT may, however, not provide conservative models because it may under-predict risk, especially when significant site blocking, NM detachment and/or size exclusion occur. Size exclusion, in particular, may lead to very fast spreading of NMs in the soil profile. It therefore remains to be investigated how prevalent these mechanism are, preferably in realistic systems using low concentrations and a relevant exposure scenario. Only one study with NMs on undisturbed columns is available, where only a blocking mechanism was needed to explain high transport of AgNP [41].

Finally, speciation may be an important mediator of toxicity, but the above transport modelling does not predict NMs’ speciation and transformations, i.e., the final form in which the NM occurs in soils. Moreover, the history of NMs may be decisive in transport modelling, for example NMs entering soils together with biosolids do so coated with organic materials or hetero-aggregated with other particles [17]. If these interactions are irreversible, they have a profound effect on how NMs are transported in soils relative to a well-defined case of sand columns exposed to well-dispersed NM suspensions. Soil transport models may therefore have to be combined with models developed for aquatic compartments to predict NMs speciation as well. A further discussion of the possible way forward in fate assessment of NMs can be found in [51]. If exposure can definitely be excluded, no hazard assessment has to be performed (Phase 1) to evaluate the risk from a scientific point of view. 

### 2.3. Hazard

**Figure 7:**
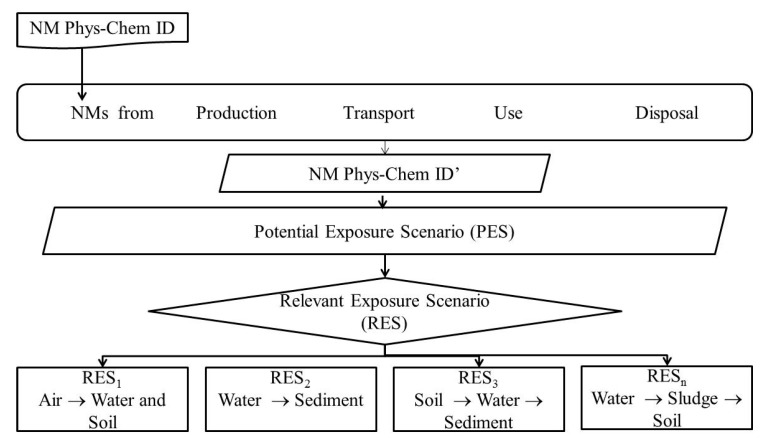
Identification of Relevant Exposure Scenarios (RES) based on identified release along the NMs life cycle, the NMs characteristics at each point, and the Potential Exposure Scenarios for such release.

With the identified PES and RES (Figure 7), potentially affected organisms are defined within a representative group of organisms in order to account for the bio-diversity in each environmental compartment. 

The appropriateness of the OECD test guidelines as well as other guidelines for NMs has been reviewed [52,53]. In the OECD document [52], it was concluded that the majority of OECD test guidelines are applicable to NMs, however, carefully noting that “the guidance on preparation, delivery, measurement, and metrology is currently insufficient for testing of manufactured nanomaterials”. Based on the preliminary guidance notes from 2010, the OECD published the additional guidance document: “Guidance on sample preparation and dosimetry” in 2012 (see Guidance on Sample Preparation and Dosimetry for the Safety testing of Manufactured Nanomaterials. ENV/JM/MONO(2012)40). In the final project report for the REACH Implementation Projects on Nanomaterials (RIP-oN 2), part 2, it is concluded for the 31 OECD test guidelines for the determination of potential ecotoxicological effects that “Thus, the basic toxicological properties as well the endpoints described and determined in these guidelines are adequate and relevant also for nanomaterials” [54].

Based on the discussions during the OECD expert meeting on ecotoxicology and environmental fate [55], several OECD member countries engaged in the “OECD Working Party on Manufactured Nanomaterials” initiating the update of specific existing OECD test guidelines for NMs and the drafting of new ones. The OECD test guidelines are methods for regulatory testing of chemicals, and data generated by these test guidelines fall under the agreement on Mutual Acceptance of Data (MAD) in the Assessment of Chemicals (OECD 1981, see http://www.oecd.org/env/ehs/mutualacceptanceofdatamad.htm) and are recognised in countries adhering to MAD, which is, hence, an essential component for international harmonisation of approaches to chemical safety. Thus, the OECD is interested in ensuring that OECD test guidelines are applicable to NMs, thereby falling under MAD. OECD guidance documents on testing do not fall under MAD, but nevertheless reflect an agreement on best available procedures.

**Figure 8:**
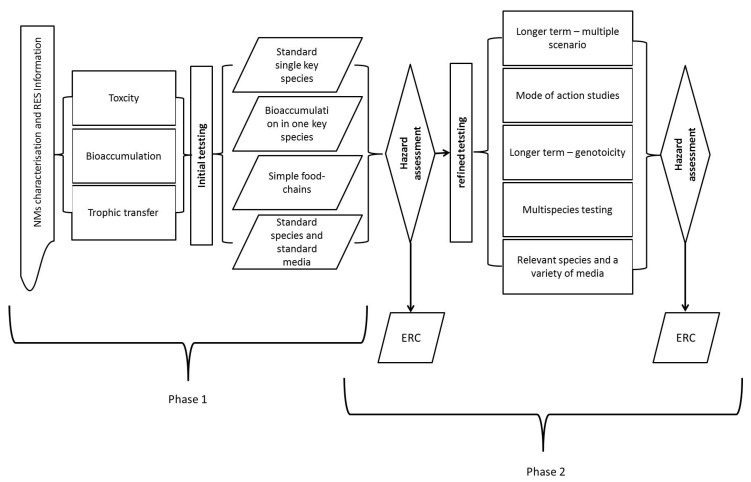
Schematic representation of the hazard testing regime for NMs proposed in MARINA, covering initial (always required) testing and refined testing. RES = Relevant Exposure Scenario; ERC = Environmental Risk Characterisation.

The present hazard testing, as outlined in [6], is divided into initial and refined testing. Through trigger values, the initial testing indicates whether refined testing is required (see Figure 8). The conventional endpoints tested in the regulatory OECD test guidelines and used for risk assessment are selected to protect environmental populations and cover parameters such as reproduction, mortality and growth. As suggested by [54], “… the basic toxicological properties as well the endpoints described and determined in the guidelines are adequate and relevant also for nanomaterials”, although there may be important modifications in the most sensitive parameters compared to chemicals. Besides the regulatory test guidelines, researchers continuously propose and publish alternative test methods and endpoints for the assessment of NMs (e.g., [53]). These alternative tests usually address mechanistically based responses (e.g., determination of specific enzymes or gene activities), which often increase sensitivity and enable an understanding of how NMs cause toxicity, often embedded in Pathways of Toxicity (PoT). It is not always obvious whether an effect detected by a sensitive additional test has an adverse effect on the organism or population studied. However, such additional endpoints can be linked to population level via Adverse Outcome Pathways (AoP, [56,57]). Several studies have indicated that metal based NMs may indeed have a different PoT and AoP compared to free ions of the same metal (see e.g., [58,59]). A possible way forward on how to include novel tools and AoP/PoTs has been discussed in [57], and discussion as to how this is linked to fate assessment is provided in Scott-Fordsmand et al. [60]. Hence, these additional test parameters (endpoints) can provide valuable information on ecotoxicity of NMs that support the hazard assessment, which is important in particular for read-across issues, i.e., read across between organisms and between materials. In fact, it is likely that the current lack of agreed mechanistic based endpoints for regulatory purposes has inhibited the derivation of read-across between species and materials. For example, although it is known that some chemicals/materials cause toxicity via the same mechanism, this can and has only been identified using environmental test systems different from the OECD test guidelines. It is, thus, important to note that research on the suitability of alternative endpoints is ongoing, but final conclusions or derivation of validated test guidelines are not yet possible. In any case, in research alternative endpoints play a major role by increasing the knowledge on the mode of action of NMs and improving their hazard assessment. Furthermore, little is known regarding whether there are specific effects that are not detected within the conventional testing endpoints or timeframes, but which may have an impact on the population level and, as such, be relevant for determining the hazard of the NMs. 

The NMs’ physicochemical characteristics have also been shown to determine the uptake and toxicity. For example, materials with varying surface charge may be taken up differently for similarly sized materials, similar “sized” materials with unlike shapes e.g., rods, spheres, plates, cubes, triangles can also be taken up differently depending on local conditions, and depending on the size NMs composed of same elements may be taken up differently, (see e.g., [61,62]). These previous factors influence not only uptake and toxicity, they also influence oxidation and release from the nanomaterials. Hence, basic NM characteristics (e.g., size/shape, surface change, dissolution) are important for the hazard, as it is for exposure. 

Although the organisms used for regulatory testing of conventional chemicals are presumed to be representative to many ecosystems, little is known about this issue in the specific case of NMs. Commonly used testing organisms may not be representative of the specific environmental compartment to which NMs partition, nor may the organisms be representative if NMs have a particular mode of action. For example, for sediments it cannot be excluded that organisms living and grazing on the sediment are exposed to a higher extent compared to the standard test organisms. In the reviewed literature, mussels (*Mytilus galloprovincialis*) were commonly used as test organisms to study the ecotoxicity of TiO_2_, carbon black, fullerene and SiO_2_ [63], which is in line with other authors also studying NMs’ effects on mussels (e.g., [64,65]). Additionally, snails such as *Physa acuta*, *Lymnaea stagnalis* or *Pyringa ulvae* might be of interest, since they are sediment feeders (e.g., [66,67]); these latter organisms do not belong to the current OECD test organisms. Relevant organisms that are already used for the assessment of chemicals are amphipods such as *Hyalella azteca* and *Gammarus pulex*. Generally, also for soils and water compartments, information on the sensitivity of alternative organisms compared to the traditional ones is very limited, and such issues should be studied for each of the relevant environmental media. An additional important issue for the current test systems is whether the duration of the exposure is representative also for NMs. Since NMs may persist in the environment and can be taken up relatively slowly (compared to free ions), it is likely that toxicity is only expressed in the longer term, hence, long-term test systems should be applied. Further, it is still under discussion whether homogenous and stable test dispersions of NMs, obtained by using stabilizers, have to be used, or whether mechanical dispersion reveals itself to be unsuitable due to a high sensitivity of the test organism (e.g., daphnids) to sedimentation of the material [68]. 

In order to determine the environmental hazard, which is different from the toxicity to the individual species, the Predicted No Effect concentration [69] is estimated. For this, two general approaches have been used: a deterministic and a probabilistic based approach. The deterministic approach is a factorial approach in which assessment factors are considered for chemicals (see Table 2). This approach is also considered for NMs, obviously necessitating further consideration of how far the assessment factors cover NMs. Since the factors applied to chemicals in general are arbitrarily chosen to yield conservative (safe) values and are based on little experimental evidence, the applicability of the current factors for chemicals also to NMs is simply a choice. Nevertheless, ECHA has evaluated that the assessment factors are suitable [70] and guidance is given on assessment factors for each environmental compartment, see https://echa.europa.eu/documents/10162/13632/ information_requirements_r10_en.pdf. The validity of this approach could be confirmed, or the opposite, though an analysis of the sensitivity range for organisms to various chemicals versus the sensitivity range to NMs could be performed. However, since at present very few NMs have been tested, such a comparison would not result in additional insights. Finally, weight of evidence approaches may also be considered when little information is present.

Furthermore, as described in the life cycle and release sections, a NM may change during its lifecycle, which obviously complicates the hazard assessment. If it can be scientifically argued that if all nano-forms (i.e., different forms of the pristine NM) of the substance demonstrate the same exposure and eco-toxicological profile, then the deterministic evaluation in the PES may be sufficient (e.g., for REACH registration). However, if evidence indicates that particular nano-forms have different hazard profiles, then it would be appropriate to include the Phase 2 RES approach within an evaluation and registration. 

**Figure 9:**
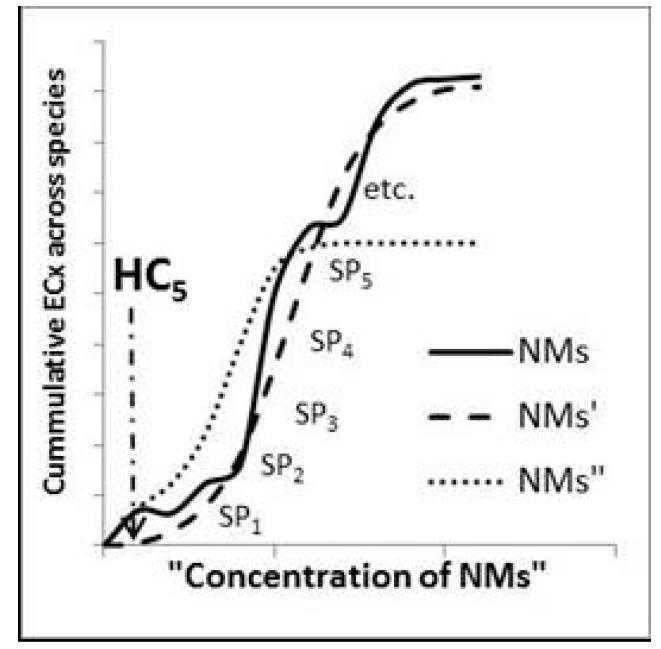
Illustration of the probabilistic based approach to derive PNEC, where cumulative curves are built over the toxicity data and a Hazard Concentration 5 (HC5) is statistically derived. NMs, NMs′ and NMs′′ refer to the changes in NM form during different stages of the life cycle. SP_1_, SP_2_, etc. refer to different species.

For the refined hazard assessment, a probabilistic based model should be used (see Figure 9). For the distribution based approaches, these have been based on various basic assumptions (see e.g., [71,72]), with the latest development that includes weighted data input [73]. Examples of use for the Species Sensitivity Distribution (SSD) approach for NMs can be found in [72,74,75]. The SSD approach refers to techniques where there is an assumption that the sensitivity (given as an ECx values) of a number of species can be modelled to fit a distribution, and using this an ECx for the most sensitive species can then be derived with a statistical certainty. Common for all of these probabilistic models is that they require input data representative of the problem frame and that an increase in the number of input data reduces the quantifiable uncertainty (the latter is in sharp contrast to the deterministic approach). With the most recent development of a weighted approach, it is possible to introduce factors beyond individual toxicity data, e.g., by taking NM physico-chemical parameters into account.

### 2.4. Risk Characterisation

As mentioned in the beginning of the paper, the ERA approach for NMs is an exposure driven process comprising two general phases, as outlined by [6], covering the whole life cycle of the material. The Environmental Risk Characterisation depends on the predicted environmental concentration (PEC), the effect concentration (PNEC), and the relationship between these. The aim of Phase 1 is to identify Relevant Exposure Scenarios (RES), among Potential Exposure Scenarios (PES), throughout a NM′s life cycle (see Section 2.1) and to clarify what information is required in order to evaluate whether exposure in these scenarios may lead to environmental risks (Phase 2). 

**Figure 10:**
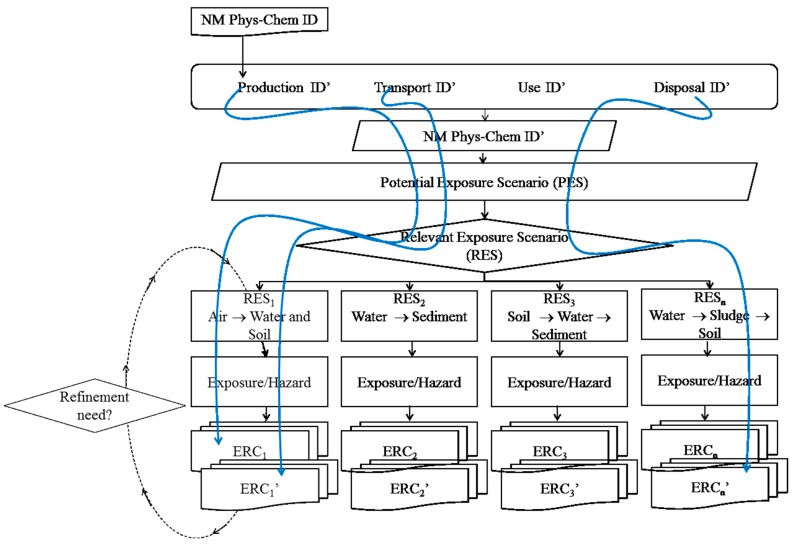
The Overall environmental Risk Assessment flow chart with examples of how one initial NM may result in multiple different (blue arrows) risk characterisations.

Phase 1 constitutes basic information that should be available for all materials and should outline the major further information requirements for Phase 2. The aim of Phase 2 is to provide a refined ERA, based on the identified RES and on the identified information requirements (Figure 10). 

In the initial data gathering process, it is obviously important to ensure data quality, i.e., the representativeness, the relevance and the reliability of the data. Various approaches to address this issue for NMs have been used and suggested, e.g., the evaluation criteria applied in previous and current ERAs, nano-specific evaluation approaches, (e.g., [76]), and weight-of-evidence based approaches, (e.g., [20]); although it is important, this issue will not be further dealt with here. For both phases, the evaluation paradigm will be similar, although more data intensive in Phase 2 than in Phase 1. 

For Phase 1, it is likely that the information level will mainly allow for a deterministic evaluation based on total media concentrations. This evaluation may be fully deterministic (based on single PEC and PNEC values), or a semi-quantitative assessment (e.g., the latter as a kind of risk banding). Similar to conventional chemicals, the PNEC may be determined by applying assessment factors (see Table 2) to the hazard information. The risk related knowledge in this phase is likely especially limited concerning the connection between material, exposure and hazard, but within the evaluation steps fate, exposure and hazard nano-relevant/-specific issues may be considered, e.g., release potential, determining particle persistence versus ion-release, and general nano-related exposure and hazard information (see e.g., Tier I-V in Collier et al. [4]). Further information on materials, exposure and hazard specific information will lead to a Phase 2 evaluation. The outcome of Phase 1 will be a risk ration value for each RES, the possibility of designing good risk mitigation measures or using this information to design safe nanomaterials based on this Phase 1 information is probably limited. It is clear that the risk estimated in the ERC may be composed of a set of different risks, depending on the material characteristics at each stage of the life cycle, for example if a material changes one or more of its characteristics (e.g., size) during the life cycle, then the ERC will represent different risk “scenarios” (see Figure 10). It is also conceivable that for a given media, e.g., soil, the ERC may differ depending on the release source along the NMs life cycle. If a property of the NMs changes during the life cycle of the material, this may influences the exposure and the hazard and, hence, the risk. Obviously, this provides the starting point for risk management, identifying areas where the largest risk reductions can be made.

Within the chemical risk assessment strategy, the hazard is defined to be an intrinsic property of the substance. Hence, for each chemical it is only possible to reduce risk further by reducing exposure. However, to some extent, NMs may display properties that can be viewed as different from the “bulk” substance properties. Different forms of the NM can display differing physicochemical characteristics, which in turn may lead to differing hazard properties, and, if possible application-wise, a careful selection of the safest NM form could also lead to reduced hazard. 

For Phase 2, the approach should mainly follow a probabilistic evaluation of the risk, e.g., based on a probabilistic environmental fate model (see Section 2.2.2), on total or bioavailable concentrations when possible (see Section 2.2.3), and on a probabilistic evaluation of a broad set of hazard data. Here, novel probabilistic approaches should be included, e.g., Monte Carlo permutation based approaches [25] and the weighted species sensitivity distribution [73]; the latter can include further differences in the input data. Since it is well known that NMs’ fate, exposure and toxicity depend on the media characteristics, the aim is to move away from generic approaches (Phase 1) into media dependent ERC. In contrast to Phase 1, it is likely that the NMs’ characteristics can be used to enable risk mitigation, e.g., by relating changes in NMs’ characteristics to changes in fate or hazard. As for Phase 1, the ERC for materials along the material’s life cycle may represent different risks, since the material may have changed. However, in Phase 2 there should be sufficient information for modelling connection(s) between these, and with the iterative approach in Phase 2 this obviously can end up in site specific assessment of the risk.

## 3. Conclusions

This paper outlines the general issues for Environmental Risk Assessment (ERA) of nanomaterials (NMs), showing that although mass may be a final concentration-metric in risk characterisation, it is vital to have many different physico-chemical descriptors (e.g., mass, number of particles, surface area, charge) for the individual NMs. It is further shown how progress is made in the area of novel NM relevant or specific fate and exposure models, and how hazard testing needs to consider alternative approaches. Finally, previously used risk characterisation models, e.g., species distribution models, have been tested for NMs identifying the challenges also in this area, e.g., how to include other physico-chemical parameters within the models. The proposed Environmental RA framework reduces the uncertainty in relation to assessment of NMs since it incorporates well established conceptual risk framework models with state of the art knowledge based guidance for the individual sub domains, i.e., material, fate, exposure, hazard and risk characterisation. Although uncertainty is reduced by the suggested approach, major uncertainties remain on all levels. Some of these will not be resolvable (and are no resolved in current chemical risk assessment either), while many can be reduced with further research in understanding which key material parameters determine the fate, exposure and toxicity for these materials. It is likely that in the long run, this is done most effectively and least costly through a mechanistic understanding of the problem, i.e., understanding the kinetics involved.

## Figures and Tables

**Table 1:** Equations (examples) used for transport modelling of different NMs in sand columns or stacked columns of natural soils.

Mechanism	Sand Columns	Natural Soils
Attachment	ρ(dS_att/str_/dt) = k_att/strr_θψC	
		Ag [39,41]
	C_60_ [42,43]	C_60_ [42,44]
		CNT [45]
Colloid filtration theory	K_att_ = α_att_((3(1−θ))/(2*d*_50_))η_0_μ	
	B [46]	Ag [39]
	CeO_2_ [47]	TiO_2_ [40]
	CNT [48]	
	C_60_ [49,50]	

ρ: dry bulk density of the packed column; S_att/str_: NM concentrations in the attachment/straining site; t: time; k_att/str_: attachment/straining rate constant; θ: porosity; ψ: blocking/straining coefficient; C: concentration of NMs in aqueous phase; α_att_: attachment efficiency; *d*50: average soil grain diameter; η_0_: single-collector deposition efficiency; μ: pore flow velocity.

**Table 2:** Example of Assessment factors for chemicals currently used in REACH (EU) for deriving Predicted No Effect Concentrations (PNEC) for assessing the Soil compartment, see https://echa.europa.eu/documents/10162/13632/information_requirements_r10_en.pdf, Table R.10-10 f. It is unknown whether these also will be adequate for NMs.

Available Information per Scenario	Assessment Factor
LC_50_ short-term toxicity test(s) (e.g., plants, earthworms, ort microorganisms)	1000
NOEC for one-long-tem toxicity test (e.g., plants)	100
NOEC for additional long-term toxicity tests of two trophic levels	50
NOEC for additional long-term toxicity tests for three species of three trophic levels	10
Species sensitivity distribution (SSD method)	5–1, to be fully justified on a case-by-case basis (cf main text)
Field data or model ecosystems	Case-by-case

LC_50_: Lethal Concentration 50%, NOEC: No observed Effect Concentration.
